# An investigation on the respiratory mechanics of mechanically ventilated patients during spontaneous breathing trials with enhanced low‐level pressure support ventilation

**DOI:** 10.1111/crj.13618

**Published:** 2023-05-09

**Authors:** Baozhu Zhang, Zhe Zhang, Haiping Qin, Zhenjie Jiang, Qiuxue Deng, Qingwen Sun, Yingzhi Wang, Jing Zhou, Zhimin Lin, Weiqun He, Dongming Hua, Yuanda Xu

**Affiliations:** ^1^ Department of Critical Care Medicine The First Affiliated Hospital of Guangzhou Medical University Guangzhou China; ^2^ Guangzhou Respiratory Health Research Institute The First Affiliated Hospital of Guangzhou Medical University Guangzhou China; ^3^ Department of Pulmonary and Critical Care Medicine The Second People's Hospital of Fengkai Zhaoqing China; ^4^ Department of General Medicine Bunbury Regional Hospital Bunbury Western Australia Australia

**Keywords:** inspiratory trigger delay, patient–ventilator asynchrony, positive end‐expiratory pressure, pressure support ventilation, spontaneous breathing trials

## Abstract

**Introduction:**

Low‐level pressure support ventilation (PSV) is most commonly adopted in spontaneous breathing trials (SBTs), and some have proposed setting the positive end‐expiratory pressure (PEEP) to 0 cmH_2_O in order to shorten the observation time of SBTs. This study aims to investigate the effects of two PSV protocols on the patients' respiratory mechanics.

**Material and method:**

A prospective randomized self‐controlled crossover design was adopted in this study, which involved enrolling 30 difficult‐to‐wean patients who were admitted to the intensive care unit of the First Affiliated Hospital of Guangzhou Medical University between July 2019 and September 2021. Patients were subjected to the S group (pressure support: 8 cmH_2_O, PEEP: 5 cmH_2_O) and S1 group (PS: 8 cmH_2_O, PEEP: 0 cmH_2_O) for 30 min in a random order, and respiratory mechanics indices were dynamically monitored via a four‐lumen multi‐functional catheter with an integrated gastric tube. Among the 30 enrolled patients, 27 were successfully weaned.

**Result:**

The S group showed higher airway pressure (Paw), intragastric pressure (Pga) and airway pressure–time product (PTP) than the S1 group. The S group also showed a shorter inspiratory trigger delay, (93.80 ± 47.85) versus (137.33 ± 85.66) ms (*P* = 0.004); and fewer abnormal triggers, (0.97 ± 2.65) versus (2.67 ± 4.48) (*P* = 0.042) compared with the S1 group. Stratification based on the causes of mechanical ventilation revealed that under the S1 protocol, patients with chronic obstructive pulmonary disease (COPD) had a longer inspiratory trigger delay compared to both post‐thoracic surgery (PTS) patients and patients with acute respiratory distress syndrome. Despite providing greater respiratory support, S group led to significant reductions in inspiratory trigger delay and less abnormal triggers compared to S1 group, especially among patients with chronic obstructive pulmonary disease.

**Conclusion:**

These findings suggest that the zero PEEP group was more likely to induce a higher number of patient–ventilator asynchronies in difficult‐to‐wean patients.

## Graphical abstract

Low‐level pressure support ventilation (PSV) is most commonly adopted in spontaneous breathing trials (SBTs). This study aims to investigate the effects of two PSV protocols on the patients' respiratory mechanics. These findings suggest that the enhanced low‐level PSV protocol was more likely to induce a higher number of patient–ventilator asynchronies in difficult‐to‐wean patients.

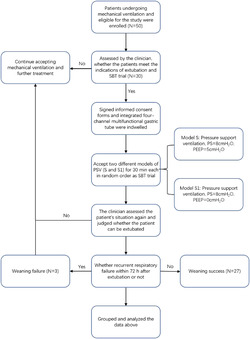

## 1 INTRODUCTION

Invasive mechanical ventilation is an important intervention for patients with respiratory failure, in which the weaning process accounts for 42% of total mechanical ventilation time.^1^, ^2^ Beginning weaning too early may cause excessive fatigue of the respiratory muscles, which can easily lead to weaning failure, whereas weaning too late may result in more complications related to mechanical ventilation, ventilator dependence and higher mortality rates.^3^, ^4^ However, there is currently no gold standard for clearly determining the success or failure of weaning, and clinicians can only reach a comprehensive judgement by monitoring the patient's respiratory function and various indicators.

The 2017 American Thoracic Society guidelines for weaning critically ill patients from mechanical ventilation^5^ recommend that spontaneous breathing trials (SBTs) should be conducted on patients who have been mechanically ventilated for more than 24 h. SBTs are commonly employed by clinicians to predict the success or failure of weaning in patients, and techniques include low‐level pressure support ventilation (PSV), continuous positive airway pressure (CPAP) and T‐piece.^3^ At present, low‐level PSV is commonly adopted for SBT, as it has the advantages of a high pass rate, high extubation success rate and a tendency towards reducing intensive care unit (ICU) mortality.^5^, ^6^ However, controversy remains over the SBT. First, because low‐level PSV offers the greatest respiratory support, there are doubts as to whether patients will be able to successfully re‐establish spontaneous breathing after being weaned. Second, the duration of SBTs is also a topic of debate^7^, ^8^; the procedure for conventional SBTs involves an initial observation of 3 min after implementation, which is continued for another 30 min if successful. There have been some cases where the observation time was extended to 2 h or longer, which were related to the patient's underlying condition, age and invasive ventilation time. However, an excessively long SBT time can easily lead to respiratory muscle fatigue, which will increase the likelihood of weaning failure or reintubation.

Previous studies have found that T‐piece ventilation was more demanding than PSV, and although the former is closer to the respiratory load after weaning, its prolonged implementation can easily lead to ventilator fatigue. The standard low‐level PSV method recommends administering 5–8 cmH_2_O of PSV or automatic tube compensation^6^ but does not specify the timing involved. Thus, in order to identify an SBT time window that is more in line with clinical needs, Subirà et al^9^ found that compared to T‐piece ventilation, low‐level PSV without PEEP led to higher rates of weaning success and similar reintubation rates. Therefore, what are the effects of a low‐level PSV protocol on the respiratory mechanics of difficult‐to‐wean (DTW) patients? In this study, DTW patients underwent two different 30‐min SBT protocols: (1) the standard low‐level PSV protocol (PS: 8 cmH_2_O, PEEP: 5 cmH_2_O; referred to as S group) and (2) the enhanced low‐level PSV protocol (PS: 8 cmH_2_O, PEEP: 0 cmH_2_O; referred to as S1 group). The effects of these PSV protocols on the patient's respiratory mechanics were compared in order to determine the appropriate parameter settings for SBTs. In addition, the patient's respiratory mechanics were continuously monitored via an indwelling four‐lumen multi‐functional catheter with integrated gastric tube, which prevented the disruption of routine treatment and greater invasiveness of placing a single oesophageal manometry catheter.

## 2 MATERIALS AND METHODS

### 2.1 Patients

A prospective, self‐randomized crossover study was conducted in the ICU of the First Affiliated Hospital of Guangzhou Medical University between July 2019 and September 2021.The inclusion criteria were as follows: (1) patients aged 18–70 years; (2) DTW patients who have received mechanical ventilation for more than 48 h; and (3) patients who have been withdrawn from sedatives, had plans to undergo SBT again and for whom the S/S1 group was selected. The exclusion criteria were as follows: (1) patients with neuromuscular disease; (2) patients diagnosed with unilateral/bilateral diaphragmatic dysfunction; (3) patients with high cervical spinal cord injury; and (4) delirious or uncooperative patients.

### 2.2 Methods

Demographic data (age, sex and ethnicity), profiles of comorbidities and hospital admission parameters (symptoms and time of symptom onset related to difficult weaning, vital signs and laboratory tests) were obtained by authors. A prospective randomized self‐controlled crossover design was adopted in this study. Patients undergoing weaning who were enrolled for SBT observation received the placement of a gastric tube with integrated four‐lumen multi‐functional catheter. The pressure extension tubes of the oesophageal and gastric balloons were connected to the Validyne DP15 pressure sensor (USA). The ventilator circuit was also connected to the Validyne DP15 through pressure extension tubes via a Y‐shaped connector. The three‐channel pressure signals were amplified using a Validyne CD280 pressure amplifier. After adjustments to notch and gain, the pressure were imported into Powerlab and then exported to LabChart 8 for data acquisition.

Patients underwent 30 min of either the S group or S1 group in a random order. During SBT, the patient's fraction of inspired oxygen (FiO_2_) remained unchanged. At the end of SBT, collect the following ventilator‐related indicators: respiratory rate (RR), rapid shallow breathing index (RSBI), inspiratory time (Ti), expiratory time (Te), total time‐over‐threshold (Ttot); airway pressure (Paw), esophageal pressure, and intragastric pressure (Pga) were recorded for 2 min. Five consecutive breathing cycles were analysed using LabChart 8 to calculate the transdiaphragmatic pressure (Pdi), airway pressure–time product (PTP), oesophageal pressure–time product (PTPes), intragastric pressure–time product (PTPga), transdiaphragmatic pressure–time product (PTPdi), ratio of transdiaphragmatic to oesophageal pressure–time product (PTPdi/PTPes) and ratio of oesophageal to transdiaphragmatic pressure (Pes/Pdi). The inspiratory trigger delay was calculated based on the time difference between inspiratory onset and ventilator flow delivery determined using oesophageal pressure, the frequency of abnormal triggers within 2 min was calculated, and the asynchrony index was calculated using the central respiratory frequency within 2 min.

**FIGURE 1:**
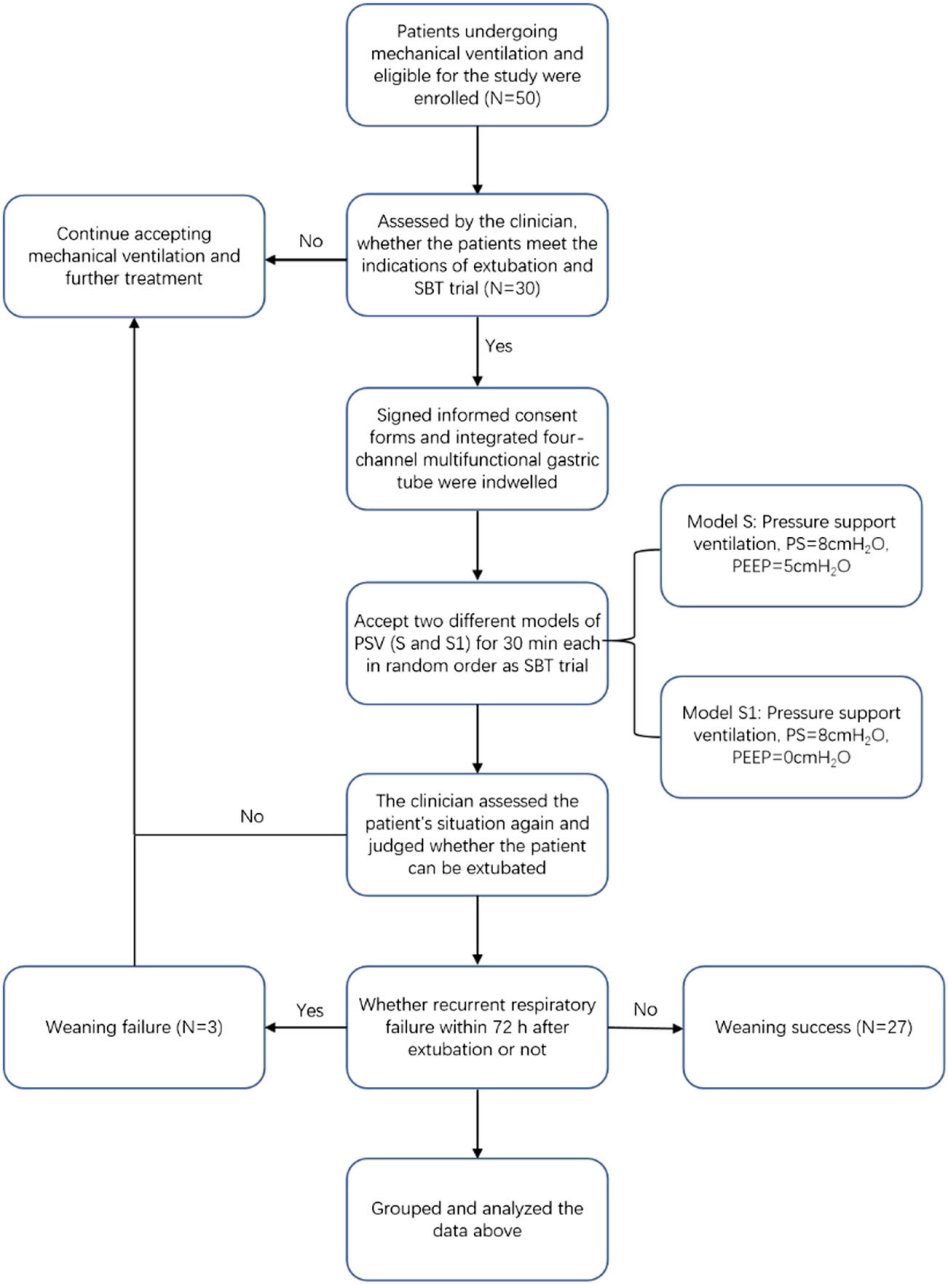
Flow chart. PEEP, positive end‐expiratory pressure; PS, pressure support; PSV, pressure support ventilation; SBT, spontaneous breathing trials.

Patients who successfully completed the SBT were assessed by the attending physician to determine whether they can undergo extubation. Patients who could not tolerate the SBT continued receiving ventilation with adjustments made to the pre‐SBT parameters. The criteria for SBT failure were as follows: RSBI >105, respiratory rate (RR) <8 or >35 breaths/min, spontaneous breathing tidal volume<4 mL/kg, heart rate (HR) > 140 beats/min or changes >20% and occurrence of new‐onset arrhythmia. Patients who developed respiratory failure within 72 h after extubation were reintubated. Weaning success was defined as the absence of reintubation 48 h after extubation (see Figure 1).

## 3 STATISTICAL ANALYSIS

Data processing was performed using SPSS 21.0. General data that were normally distributed were expressed as mean ± standard deviation (x ± s), and those that were non‐normally distributed were expressed as median (lower quartile, upper) [M (QL, QU)]. Paw, Pes, Pga and other data under the S and S1 modes were subjected to paired *T* test and non‐parametric Wilcoxon test. After stratification based on different causes of mechanical ventilation, normally distributed data were subjected to one‐way analysis of variance (ANOVA), whereas non‐normally distributed data were subjected to non‐parametric Kruskal–Wallis (KW) test. Data stratified by weaning outcome were analysed using binomial logistic regression. *P* < 0.05 indicated statistical significance.

## 4 RESULTS

A total of 30 DTW patients who had received mechanical ventilation for more than 48 h were enrolled, 27 patients with successful weaning, all patients tolerated the entire duration of the S mode, whereas three patients could not tolerate the S1 mode. See Table 1 for general data.

**TABLE 1:** Demographics and baseline characteristics of enrolled patients.

Characteristics	Value
Age, years	62.50 (53.5, 69)
Females (%)	8 (27%)
Predicted body weight (PBW), kg	58.50 (54.00, 63.00)
Body mass index, kg/m^2^	21.00 (18.00, 24.00)
APACHE II	18.00 (14.50, 24.50)
SOFA	9.00 (5.00, 12.00)
Causes of mechanical ventilation, n	
Chronic obstructive pulmonary disease (COPD)	7 (23.33%)
Post‐thoracic surgery (PTS)	13 (43.33%)
Acute respiratory distress syndrome (ARDS)	10 (33.33%)
Hospitalization time, days	35.50 (24.5, 50.75)
ICU hospitalization time, days	31.00 (13.00, 47.50)
Invasive ventilation time, days	14.00 (8.00, 26.50)
Noninvasive ventilation time, days	2.00 (2.00, 5.00)

Abbreviations: APACHE II, Acute Physiology and Chronic Health Evaluation II; ICU, intensive care unit; SOFA, The Sequential Organ Failure Assessment.

**FIGURE 2:**
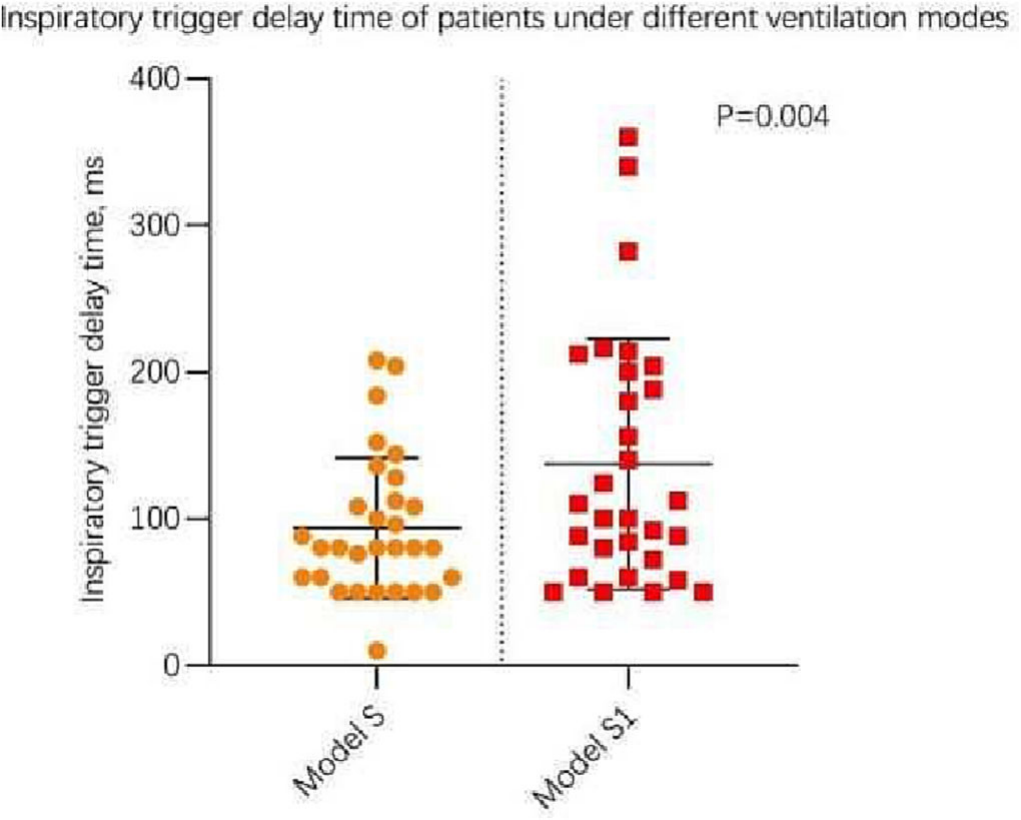
Scatter chart of inspiratory trigger delay time of patients under S and S1mode.

**FIGURE 3:**
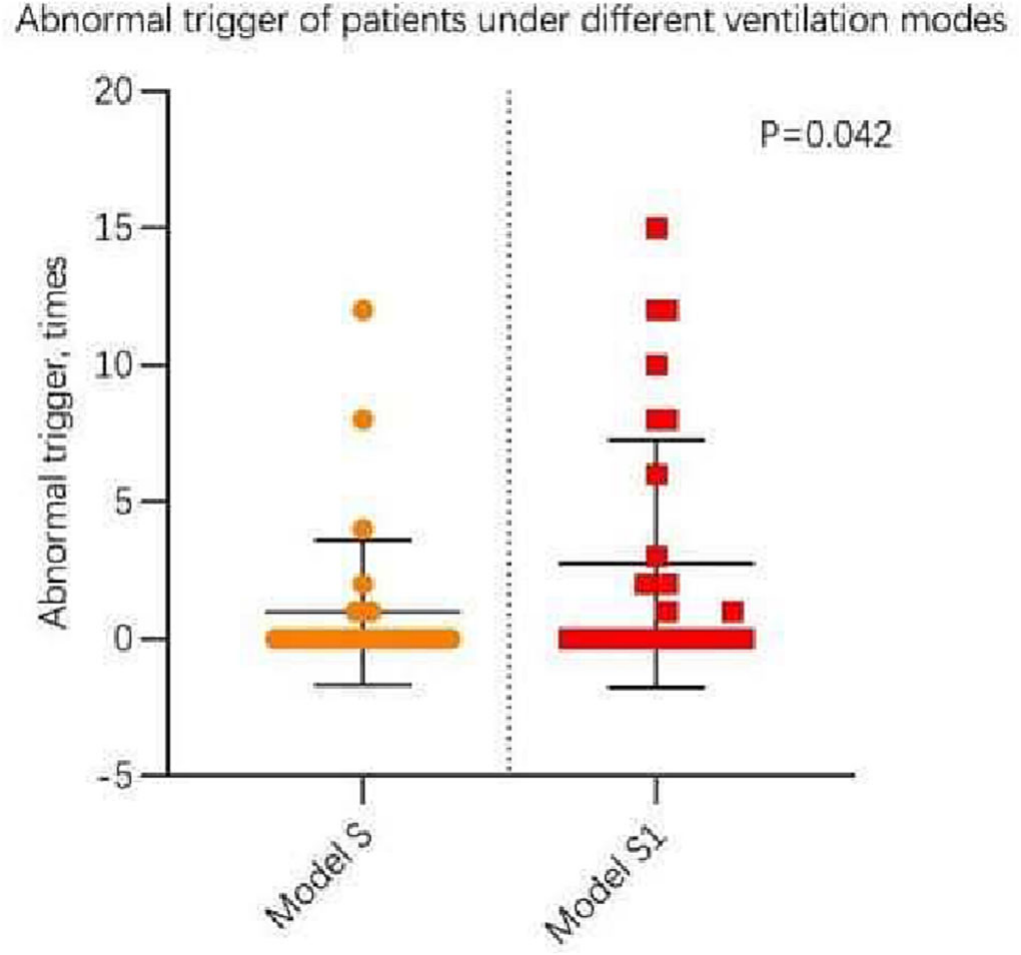
Scatter chart of abnormal trigger of patients under S and S1mode.

Table 2 shows the comparison of respiratory mechanics parameters measured under the S and S1 modes during SBT. The results indicate that the Paw Pga and PTP of the S group was higher than that of the S1 group (*P* < 0.05); meanwhile, S group has shorter inspiratory trigger delay and less failed triggers compared with S1 group (*P* = 0.004) (see Figure 2); and the S group had fewer abnormal triggers than the S1 group (*P* = 0.042) (see Figure 3). No significant differences were observed for the remaining parameters.

**TABLE 2:** Respiratory mechanics of patients under different ventilation modes.

Indices	S mode	S1 mode	P value
RR	22.27 ± 4.43	23.33 ± 5.91	0.409
Pes, cmH_2_O	5.16 ± 9.14	3.55 ± 9.71	0.053
Pga, cmH_2_O	13.94 ± 9.83	11.90 ± 8.16	0.025
Paw, cmH_2_O	11.33 ± 0.96	5.85 ± 1.25	<0.001
Pdi, cmH_2_O	9.03 ± 13.40	8.35 ± 12.24	0.705
Pes/Pdi	1.07 ± 3.72	0.28 ± 1.78	0.222
PTP, cmH_2_O·s	12.20 ± 3.06	6.40 ± 2.21	<0.001
PTPga, cmH_2_O·s	14.56 ± 10.15	13.09 ± 9.85	0.100
PTPdi, cmH_2_O·s	8.96 ± 13.87	9.82 ± 14.21	0.496
PTPdi/PTPes	−2.44 ± 20.49	−11.78 ± 66.05	0.358
Ti, s	1.07 ± 0.22	1.07 ± 0.28	0.965
Te, s	1.88 ± 0.83	1.95 ± 0.93	0.566
Ttot, s	2.95 ± 1.00	3.02 ± 1.13	0.622
Inspiratory trigger delay, ms	93.80 ± 47.85	137.33 ± 85.66	0.004
Abnormal trigger, times	0.97 ± 2.65	2.67 ± 4.48	0.042
Asynchrony index, %	4.41 ± 11.57	11.77 ± 20.12	0.084

*Note*: Data are expressed as “mean ± SD” if not otherwise specified.

Abbreviations: Paw, airway pressure; Pdi, transdiaphragmatic pressure; Pes, oesophageal pressure; Pga, intragastric pressure; PTP, airway pressure–time product; PTPdi, transdiaphragmatic pressure–time product; PTPes, oesophageal pressure–time product; PTPga, intragastric pressure–time product; RR, respiratory; rate; Te, expiratory time; Ti, inspiratory time; Ttot, total time respiratory cycle.

**FIGURE 4:**
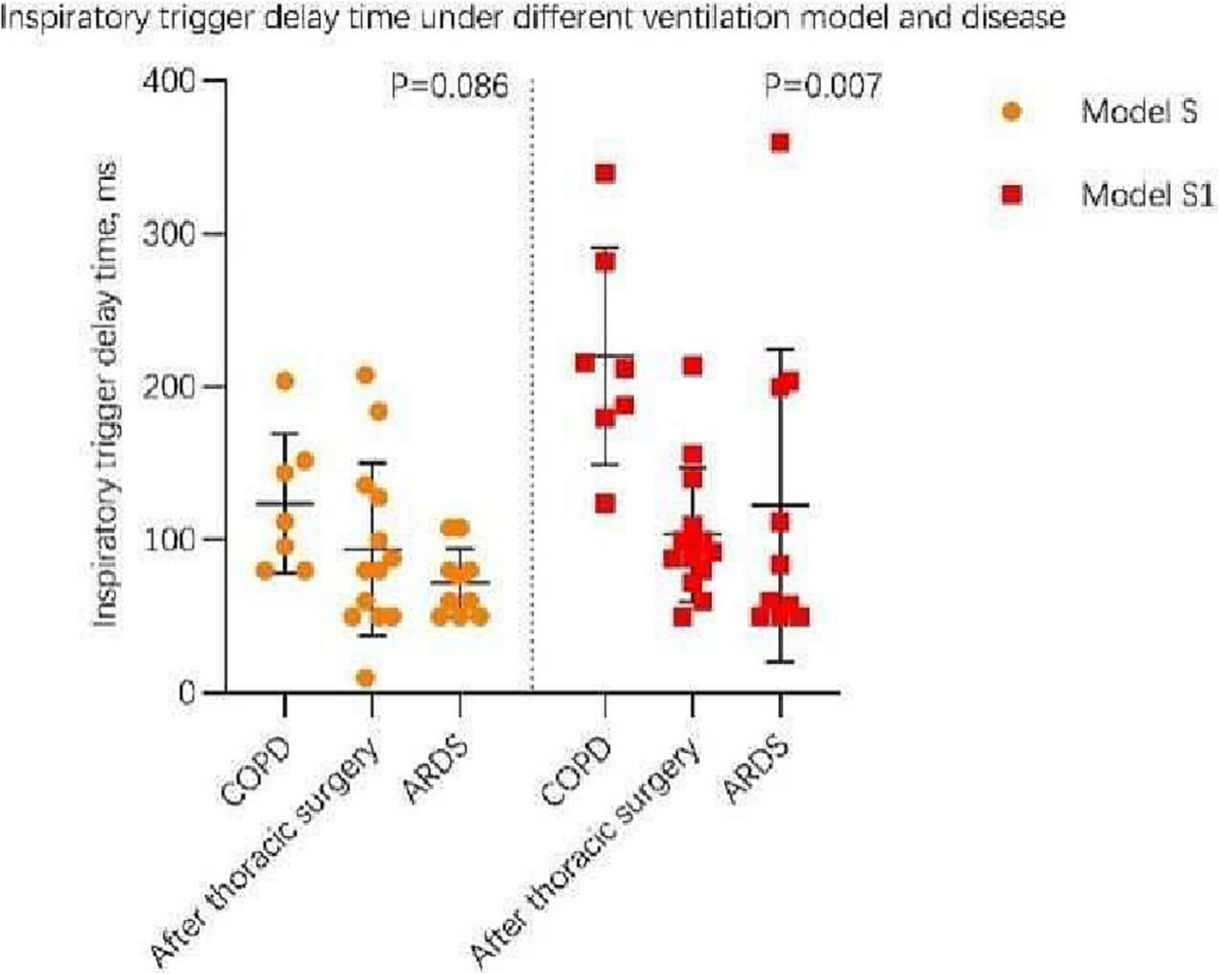
Scatter chart of inspiratory trigger delay time of patients under different ventilation model and disease. ARDS, acute respiratory distress syndrome; COPD, chronic obstructive pulmonary disease.

Table 3 shows the comparison of respiratory mechanics indices between the S and S1 modes after patient stratification based on the causes of mechanical ventilation. Under the S1 mode, COPD patients showed a longer inspiratory trigger delay compared with PTS patients (see Figure 4) and with ARDS patients (Pbetween‐group = 0.007); no significant differences were found for the remaining indices.

**TABLE 3:** Respiratory mechanics of patients under different modes and causes of mechanic ventilation.

Respiratory mechanics		Causes of mechanical ventilation			*P*			
		AECOPD (1)	PTS (2)	ARDS (3)	Overall	1 vs 2	1 vs 3	2 vs 3
Pes	S	3.77 ± 1.61	8.76 ± 12.64	1.05 ± 2.98	0.023	0.894	0.486	0.018
	S1	−3.25 (−3.83, 2.97)	3.68 (1.87, 9.28)	0.65 (−2.69, 5.62)	0.052	——	——	——
Pga	S	12.55 (8.33, 21.23)	11.74 (7.35, 24.98)	7.42 (6.59, 13.21)	0.238	——	——	——
	S1	10.70 (7.49, 15.60)	11.41 (10.90, 11.93)	11.28 (10.79, 11.46)	0.344	——	——	——
Paw	S	11.41 (11.22, 11.74)	11.41 (10.91, 11.84)	11.27 (10.92, 11.40)	0.574	——	——	——
	S1	6.16 (5.47, 6.67)	5.96 (5.74, 6.33)	6.12 (5.61, 6.65)	0.952	——	——	——
Pdi	S	12.66 ± 11.30	6.88 ± 17.68	9.32 ± 6.69	0.669	0.645	0.879	0.912
	S1	11.18 (4.52, 18.88)	7.40 (−0.29, 15.56)	4.70 (4.26, 12.54)	0.502	——	——	——
Pes/Pdi	S	0.37 (0.20, 0.74)	0.29 (−0.10, 0.88)	0.13 (−0.02, 0.54)	0.543	——	——	——
	S1	−0.17 (−0.30, 0.05)	0.17 (−0.17, 1.11)	0.31 (−0.29, 1.25)	0.367	——	——	——
PTP	S	14.05 ± 4.37	11.80 ± 2.34	11.41 ± 2.56	0.180	1.40	1.47	1.25
	S1	6.40 (4.99, 6.84)	6.10 (4.59, 6.70)	6.43 (5.67, 8.11)	0.435	——	——	——
PTPes	S	3.25 (2.84, 6.12)	5.53 (2.16, 9.93)	1.38 (−1.59, 4.25)	0.046	1.000	0.260	0.047
	S1	−2.49 (−4.17, 2.87)	12.00 (9.65, 13.12)	11.75 (9.69, 13.05)	0.054	——	——	——
PTPga	S	19.39 ± 12.20	15.82 ± 11.08	9.55 ± 4.54	0.120	0.716	0.119	0.293
	S1	15.69 ± 12.51	15.08 ± 11.27	8.68 ± 2.56	0.105	0.990	0.320	0.273
PTPdi	S	14.52 ± 11.14	6.22 ± 18.52	8.61 ± 5.08	0.456	0.425	0.682	0.918
	S1	16.52 ± 13.66	7.37 ± 18.32	8.31 ± 5.68	0.370	0.368	0.479	0.986
PTPdi/PTPes	S	2.73 (1.76, 5.11)	0.25 (−0.32, 3.39)	0.40 (−3.28, 1.84)	0.171	——	——	——
	S1	−3.19 (−4.59, 1.44)	0.36 (−0.75, 3.38)	0.48 (−3.32, 0.80)	0.615	——	——	——
Inspiratory trigger delay	S	124.00 ± 45.43	94.15 ± 56.64	72.20 ± 22.30	0.086	0.352	0.070	0.491
	S1	220.29 ± 70.90	103.85 ± 44.04	122.80 ± 102.46	0.007	0.006	0.033	0.817
RSBI	S	49.40 ± 18.45	55.64 ± 23.24	63.48 ± 14.74	0.350	0.779	0.330	0.616
	S1	56.17 ± 23.54	74.94 ± 43.78	67.39 ± 37.91	0.581	0.553	0.823	0.886

*Note*: Data are expressed as ‘mean ± SD’ if they are normally distributed, otherwise expressed as ‘median (Q1, Q3)’.

Abbreviations: AECOPD, acute exacerbations of chronic obstructive pulmonary disease; ARDS, acute respiratory distress syndrome; Paw, airway pressure; Pdi, transdiaphragmatic pressure; Pes, oesophageal pressure; Pga, intragastric pressure; PTP, pressure–time product; PTPdi, transdiaphragmatic pressure–time product; PTPes, oesophageal pressure–time product; PTPga, intragastric pressure–time product; PTS, post‐thoracic surgery; RSBI, rapid shallow breathing index.

Under the S mode, when compared with ARDS patients, PTS patients showed higher Pes (*P* = 0.018) and PTPes (*P* = 0.047); no significant differences were found for the remaining indices.

## 5 DISCUSSION

This is the first study that employed respiratory mechanics to compare different SBT modes and examine their underlying mechanism. Our findings revealed that pressure support with zero PEEP was more likely to induce a higher number of patient–ventilator asynchronies in difficult‐to‐wean patients.

Subirà et al^9^ compared the implementation of a shorter enhanced low‐level PSV protocol with that of conventional T‐piece ventilation and concluded that the former led to higher rates of weaning success and similar reintubation rates. However, further investigation is needed on the mechanisms underlying the effects of the enhanced low‐level PSV protocol on DTW patients.^10^ In our study, no significant difference was found in the SBT success rate between the S and S1 modes, and the extubation success rate after passing the SBT was up to 90%, which implies that the level of ventilator support was not the main factor affecting the SBT pass rate. Our findings revealed that the S mode showed higher Paw and PTP, suggesting that the ventilator provided greater assistance to the work of breathing. Furthermore, the higher Pga under the S mode may have been due to the effects of PEEP, which increased the patient's functional residual capacity, shifted the diaphragm downwards and increased the lower abdominal pressure while keeping the Pdi unchanged.

In the SBT study by Esteban et al^11^ and Perren et al,^12^ no difference was found between the 30‐ and 120‐min trials with respect to T‐piece trial success rate, reintubation rate, and mortality and length of hospitalization. However, Vallverdú et al^13^ demonstrated that 64% of SBT failure occurred within the first 30 min. Therefore, we speculated that the SBT duration could be set to 30 min for the majority of patients undergoing weaning. However, SBT duration is not the only factor involved. Liang et al^12^ found that DTW patients tended to have risk factors such as older age, COPD exacerbation, and chronic diseases, which increased the likelihood of SBT failure, and hence may require longer SBTs. Ferreira et al^14^ found that although NAVA could reduce trigger delays, double triggering was more common in NAVA. The reduced incidence of abnormal triggers in NAVA was similar to our observations, which suggests that although patients are able to enter the SBT phase as their condition improves their central respiratory drive, their ventilatory and mechanical coupling has not returned to the state prior to disease onset. Thus, the inappropriate increase in patient‐ventilator asynchrony and inspiratory load can easily lead to SBT failure.

In the present study, we found that patients showed longer inspiratory trigger delay and more abnormal triggers under the S1 mode (Figures 2 and 3), thus indicating the presence of more patient–ventilator asynchronies, which may lead to patient intolerance, underestimation of respiratory function in some patients and reduced weaning success rates. The appropriate application of extrinsic PEEP can be used to counteract intrinsic PEEP, overcome airway resistance, and enhance residual functional capacity.^15^ In the treatment of patients with obstructive sleep apnoea/hypopnea syndrome using non‐invasive ventilation, the application of appropriate PEEP can prevent upper airway collapse.^16^ Trigger delays can cause significant discomfort,^17^, ^18^ and studies have shown that reintubated patients exhibited more trigger delays than patients who were successfully extubated.^14^ Furthermore, COPD with lower inspiratory demand showed longer trigger delays,^19^, ^20^ especially in the presence of intrinsic PEEP.^21^ Taken together, these findings suggest that the S1 protocol (with PEEP set to 0 cmH_2_O) is not the optimal approach for SBT.

After stratification based on the causes of mechanical ventilation, we found that under the S1 mode, COPD patients showed the longest inspiratory trigger delay, followed by ARDS patients and finally by PTS patients (Figure 4). This implies that patient–ventilator asynchronies in the S1 mode were mostly due to COPD patients. Although Esteban et al^11^ found that the SBT pass rate, extubation success rate and reintubation rate after T‐piece SBT did not differ across different causes of mechanical ventilation (e.g. COPD, acute respiratory failure, etc.), Vallverdú et al^13^ found in the application of T‐piece SBT that COPD patients were most likely to develop intolerance. Furthermore, in the most recent randomized clinical trial by Subirà et al,^9^ it was found that COPD patients showed better tolerance for low‐level PSV compared to T‐piece ventilation. This was because COPD patients tended to have higher intrinsic PEEP and were more prone to diaphragm fatigue, which increased their likelihood of intolerance for the S1 mode and T‐piece ventilation, thereby resulting in SBT failure.

Despite enrolling DTW patients for this study, only a small number of cases exhibited extubation failure. Under the S1 mode, three patients with successful extubation could not tolerate the entire 30 min, whereas two patients with failed extubation could tolerate the entire S1 protocol, and one patient with failed extubation could not tolerate the entire S1 protocol. No significant differences were observed in the respiratory mechanics indices (e.g. Pes, Pga and Pdi) of these patients, thus suggesting that the S protocol, rather than the S1 protocol, could more accurately reflect the respiratory function of patients during SBTs.

Although this study was a prospective crossover self‐controlled trial, it has the following limitations. First, it has a small sample size from a single centre, and a randomized controlled trial was not performed to compare the two protocols. Second, only a few causes of mechanical ventilation that were relatively uniform were included in this study. Third, only 30‐min trials were compared between the two protocols, and the respiratory mechanics indices for 2‐h trials were not observed. Fourth, extubation was actively followed by non‐invasive mechanical ventilation or high‐flow nasal cannula oxygen therapy. Thus, although none of the patients with successful SBT were reintubated, we cannot rule out the possibility that this was due to the absence of prolonged extubation. Finally, long‐term prognosis after weaning was not observed.

## 6 CONCLUSION

When compared with pressure support with zero PEEP, support with 5 cm H2O PEEP led to higher Paw, Pga and PTP, as well as significantly reduced inspiratory trigger delay and abnormal triggers, especially among COPD patients. These findings indicate that use of zero PEEP led to more patient–ventilator asynchronies, so we do not recommend its routine implementation in DTW patients.

## AUTHOR CONTRIBUTIONS

Dongming Hua and Yuanda Xu contributed to the conception and design. Dongming Hua, Yuanda Xu, Zhenjie Jiang, Qiuxue Deng, Qingwen Sun and Weiqun He contributed to the provision of study materials or patients. Baozhu Zhang, Haiping Qin, Yingzhi Wang, Jing Zhou and Zhimin Lin contributed to the Collection and assembly of data. Zhe Zhang, Baozhu Zhang and Haiping Qin contributed to the data analysis and interpretation. All authors revised and approved the final version and agree with all aspects of the work in ensuring that questions related to the accuracy or integrity of any part of the work are appropriately investigated and resolved.

## CONFLICT OF INTEREST STATEMENT

None of the authors has any conflict of interest to disclose.

## ETHICS STATEMENT

This study was approved by the Institutional Review Board of the First Affiliated Hospital of Guangzhou Medical University (Medical Ethics Review no.: 2020‐159). Placement of the “four‐lumen multi‐functional catheter with integrated gastric tube” required the informed consent of family members in writing. This study was conducted according to the guidelines of the Declaration of Helsinki.

## Data Availability

All data generated or analysed during this study are included in this published article and supplement.
